# Effectiveness of inpatient psychosomatic rehabilitation in Germany: protocol of a longitudinal study with a waitlist control group

**DOI:** 10.1186/s12888-026-08852-x

**Published:** 2026-11-11

**Authors:** Christian Schlett, Fabian Lewandowski, Julian Mack, Marco Streibelt, Nico Seifert, Charlotte Ottenstein, Erik Farin-Glattacker

**Affiliations:** 1 Section of Health Care Research and Rehabilitation Research, Institute of Medical Biometry and Statistics, Faculty of Medicine and Medical Center, University of Freiburg, Hugstetter Straße 49, 79106 Freiburg, Germany; 2 Department of Rehabilitation Sciences, Deutsche Rentenversicherung Bund, Ruhrstr. 2, 10704 Berlin, Germany; 3 Center for Methods, Assessment and Evaluation, RPTU University Kaiserslautern-Landau, Fortstr. 7, 76829 Landau, Germany

**Keywords:** Inpatient rehabilitation, Mental well-being, Return to work, Work ability, Waiting times, Routine data, Propensity score matching, Moderator analysis, Discontinuous growth model

## Abstract

**Background:**

In Germany, psychosomatic rehabilitation is widely used to improve mental well-being and promote social and occupational participation among individuals with chronic mental and psychosomatic disorders. Although previous studies have reported clinically meaningful improvements following rehabilitation, most have relied on uncontrolled pre-post designs, limiting the strength of the current evidence. The present study exploits the current long waiting times for psychosomatic rehabilitation to form a waitlist control group without influencing the timing of rehabilitation. Overall, the primary objective of our study is to evaluate the short- and medium-term effects of psychosomatic rehabilitation on mental well-being (primary outcome), return to work, and work ability (secondary outcomes). Additional objectives include examining treatment effects in relevant patient subgroups (e.g., diagnostic groups), investigating moderator effects, and analyzing determinants of waiting time until rehabilitation.

**Methods:**

We will conduct a prospective longitudinal study with a waitlist control group among individuals whose application for inpatient psychosomatic rehabilitation has been approved by the German Pension Insurance agency (Deutsche Rentenversicherung [DRV]). Participants will complete up to 12 online questionnaires at six-week intervals over a period of 16 months. Questionnaire data will be linked with routine data from the DRV rehabilitation statistics database to obtain objective information on rehabilitation and return to work. The effectiveness of psychosomatic rehabilitation will be examined using two complementary analytical approaches: a propensity score-matched waitlist control group comparison to examine the short-term effects of rehabilitation and a discontinuous growth model to examine the medium-term effects of psychosomatic rehabilitation. Both models will also be applied in the subgroup analyses and extended by the relevant interaction terms for the moderator analyses. Determinants of waiting time until rehabilitation will be analyzed using time-to-event models.

**Discussion:**

The study will provide comparatively robust evidence on the effectiveness of psychosomatic rehabilitation in routine care. It may identify patient groups that benefit differently from rehabilitation and factors associated with waiting time until rehabilitation. These findings may inform future efforts to optimize rehabilitation interventions, improve equity in treatment outcomes, and reduce waiting times for psychosomatic rehabilitation.

**Trial registration:**

German Clinical Trials Register (DRKS): DRKS00038670 (9th December 2025; https://drks.de/search/en/trial/DRKS00038670/details); WHO UTN: U1111-1332-6419.

## Background

Mental disorders are highly prevalent in Germany and indeed across the globe, with evidence indicating increased incidence in recent years and decades [1–4]. The 12-month prevalence of mental disorders in the adult population in Germany, as estimated based on clinical interviews, is approximately 28% [5], and the incidence based on administrative data is even higher (38% [1]). These disorders, especially when chronic, have far-reaching consequences for both the social and occupational participation of those affected. In Germany, in 2024, these disorders accounted for 17% of all sick leave days (147.3 million days in total [6]) and were the most common cause of early exit from the workforce, accounting for 40% of all disability pensions granted due to reduced earning capacity (69,294 cases [7]). These individual and occupational consequences are associated with a considerable economic burden. The direct and indirect costs of mental disorders were estimated to be €146.5 billion in 2018, accounting for nearly 5% of Germany ‘s gross domestic product (GDP) [8].

Psychosomatic rehabilitation plays a crucial role in the treatment of chronic mental disorders [9], as it aims to enable affected individuals to manage their chronic condition in a self-determined manner, and to support them in maintaining occupational functioning and fulfilling their roles within family and society despite illness-related limitations [10]. In accordance with its mandate under German social law, psychosomatic rehabilitation seeks to improve the health and social participation of individuals with mental disorders. In doing so, it contributes to maintaining work ability and prolonging workforce participation among affected individuals [11]. In 2024, the German Pension Insurance (Deutsche Rentenversicherung [DRV]) alone funded about 173,000 psychosomatic rehabilitations for adults; these accounted for 18% of all medical rehabilitations, making mental and psychosomatic disorders the second most frequent indication for medical rehabilitation after orthopedic and rheumatic disorders (41%), and ahead of oncological conditions (14%) [12].

Most psychosomatic rehabilitation takes place in an inpatient setting (2024: 92% vs. 8% ambulant [13]). A meta-analysis [14], along with more recent studies, has shown that inpatient psychosomatic rehabilitation is associated with clinically meaningful improvements in psychosomatic symptoms, physical activity, and social participation (e.g. [15–18]). However, the majority of the existing studies do not include a control group, which means that the observed effects cannot be clearly attributed to the rehabilitation. Studies including a control group have merely compared different therapeutic interventions within psychosomatic rehabilitation for specific subgroups (e.g., behavioral activation vs. cognitive–behavioral therapy for patients with depressive symptoms [19], and mindfulness-based vs. relaxation-based interventions for patients with depression [20]). Consequently, there is a need for controlled studies that investigate the effectiveness of psychosomatic rehabilitation, including unselected patient populations and the full range of rehabilitation interventions as they are carried out in routine care.

The main barriers to conducting controlled studies on the effectiveness of psychosomatic rehabilitation concern ethical and legal issues [21]. Once an application for rehabilitation has been approved, patients are legally entitled to receive treatment, which makes it difficult to postpone or withdraw rehabilitation for research purposes. However, the increasing prevalence of psychosomatic disorders and the corresponding rise in demand for psychosomatic rehabilitation have led to long waiting times between approval and the actual start of psychosomatic rehabilitation (year 2021: median = 91 days; mean = 113 days), with substantial variability in waiting times (SD = 81 days; DRV Bund, internal analyses). These circumstances make it possible to include a waitlist control group (CG) without study participation influencing the timing of rehabilitation. This natural waitlist CG can be made comparable to the intervention group (IG) by applying well-established statistical matching procedures to balance potential confounding variables. Taken together, the current waiting times provide an opportunity to overcome a major limitation of previous studies (the lack of control groups) and make it possible to estimate the absolute effectiveness of psychosomatic rehabilitation with comparatively strong evidential support.

Hence, the first and primary goal of this study is to examine the short- and medium-term effects of inpatient psychosomatic rehabilitation on mental well-being (primary outcome), return to work, and work ability (secondary outcomes) in routine care. With respect to these outcomes, we expect psychosomatic rehabilitation to lead to clinically relevant improvements; therefore, we postulate that patients in the IG, who undergo psychosomatic rehabilitation, will show clinically meaningful greater improvements in mental well-being (Hypothesis 1), return to work (Hypothesis 2), and work ability (Hypothesis 3) from pre- to post-assessment than patients in the CG, who do not undergo psychosomatic rehabilitation during the same period.

Previous research suggests that the effectiveness of psychosomatic rehabilitation may vary in line with patient-related factors such as diagnostic group [11], socioeconomic status [22], educational level [16], sick leave duration prior to rehabilitation [23], psychotherapy motivation [24], and intentions to apply for disability pension benefits [24–26]. However, since these studies did not include a control group, the evidence they provide regarding differences in the effectiveness of psychosomatic rehabilitation is limited. Furthermore, findings concerning the moderating role of some factors are inconsistent—for example, de Vries et al. [27] found that women benefited to a greater extent from psychosomatic rehabilitation than men, whereas later studies were not able to replicate this [16, 28].

Against this background, the second goal of the present study is twofold. First, the study will examine whether the effectiveness of psychosomatic rehabilitation can be demonstrated across relevant patient subgroups and outcome dimensions. To this end, subgroup analyses will be conducted for relevant patient groups—including diagnostic groups—and the effectiveness of psychosomatic rehabilitation will be evaluated not only with respect to overall mental well-being but also in terms of the disorder-specific symptom dimensions of somatoform complaints, depressiveness, and phobic anxiety. Second, moderator analyses will investigate whether the effectiveness of psychosomatic rehabilitation varies according to patient-related factors such as diagnostic group, gender, age, socioeconomic status, migration background, employment status at the time of application, prior sick leave duration, psychotherapy motivation, and intentions to apply for disability pension benefits, and contextual factors such as the length of the waiting time until the rehabilitation or the duration of the latter.

The third goal of this study is to examine factors that may influence the waiting time until the start of psychosomatic rehabilitation. In this regard, factors related to the rehabilitation application and allocation process (e.g., making use of the right to choose a rehabilitation clinic) and patient-related factors (e.g., psychotherapy motivation) will be examined. In a consensus-building process aimed at improving the situation for patients, healthcare providers, and insurers, high priority was given to further research on waiting times for psychosomatic rehabilitation [29]. By investigating determinants of waiting times, the present study addresses this research need and may help inform strategies aimed at reducing the currently long waiting times until psychosomatic rehabilitation.

## Methods

### Study design and procedure

The study employs a quantitative, prospective longitudinal design with a waitlist control group in which participants are invited to complete 12 online questionnaires at six-week intervals over a period of 16 months. The first questionnaire was sent to participants by email after they had returned their written consent to participate in the study. The following 11 questionnaires are sent to participants at six-week intervals after they have completed the first questionnaire. Since the start date of the approved rehabilitation is not known at the time of study enrollment for most participants, the measurement time points (MTPs) could not be aligned with individual start dates of the participants’ rehabilitation. However, the number of MTPs (12) and the interval between them (6 weeks) ensure that data are available for all participants shortly before, shortly after, and up to six months after rehabilitation. In addition to questionnaire data, routine data from the DRV’s rehabilitation statistics database (RSD) will be included and linked to the aforementioned participants’ questionnaire data. Participation in the study does not affect the waiting time for the approved rehabilitation; consequently, the waitlist control group is not defined at study entry but is identified after completion of the field phase using a predefined matching procedure.

### Study population

The study population consists of individuals whose application for inpatient or full-day outpatient psychosomatic rehabilitation has been approved by the DRV. Since inpatient and full-day outpatient psychosomatic rehabilitation differ primarily in whether patients stay overnight at the rehabilitation facility, and since most psychosomatic rehabilitation is provided on an inpatient basis in both settings, the term “inpatient psychosomatic rehabilitation” is used hereafter for the sake of simplicity.

### Inclusion and exclusion criteria

Eligibility for study participation requires the provision of written informed consent, being at least 18 years of age, and having sufficient German language proficiency to complete an online questionnaire. Furthermore, at least one of the questionnaires must be completed before the rehabilitation; otherwise, it would not be possible to match participants based on their pre-rehabilitation values or to determine their pre-post changes. Participants are also excluded if they have been approved special rehabilitation programs, such as rehabilitation for people with severe mental illness (Rehabilitation für psychisch kranke Menschen [RPK]) or rehabilitation for people with substance use disorders.

### Sample size

With regard to the primary outcome, namely mental well-being, we assume that psychosomatic rehabilitation will have a medium effect of Cohen’s d = 0.5, which is considered clinically meaningful [30, 31]. Given this effect size, a two-sided significance level of alpha = 0.05, and a power of 0.95, a conservative two-sample t-test indicates that at least 105 participants per group are required for the primary analysis. As this analysis will be conducted using an analysis of covariance (ANCOVA), which adjusts for the pre-rehabilitation outcome and thus provides more statistical power than a t-test, we applied an approximate formula developed by Borm et al. [32], which estimates the corresponding reduction in the required sample size, based on the proportion of variance in the post-rehabilitation outcome explained by the pre-rehabilitation outcome. Based on a study that assessed changes from before to after inpatient psychosomatic rehabilitation using the same outcome measure as the present study [33], we assume that the pre-rehabilitation outcome explains at least 40% of the variance in the post-rehabilitation outcome. According to Borm et al.’s [32] formula, using an ANCOVA reduces the required sample size to 64 participants per group ((105 * (1–0.40) + 1)), which corresponds to a total sample of 128 participants.

Furthermore, we expect there to be no sufficiently similar match for approximately 20% of the participants in the matching process; thus, a sample size of at least 160 participants (128/0.8) will be necessary for our primary analysis.

According to data from the DRV Bund for the year 2021, the median (waiting) time between approval of psychosomatic rehabilitation and its start was roughly three months (91 days, personal communication). Assuming an additional four to six weeks for the rehabilitation and an average of three weeks until the subsequent MTP, we expect half of the participants (which is sufficient for matching purposes) to have completed their rehabilitation by the fifth MTP. Thus, sample size calculations were based on the data availability at this time point.

We expect 20% of individuals who provide their informed consent not to complete the first questionnaire—for example, because of incorrectly entered email addresses or due to technical issues preventing the delivery of the invitation email. Among participants who complete the first questionnaire, we anticipate a dropout rate of 5–10% at each subsequent MTP, corresponding to a cumulative attrition of 20–40% by the fifth MTP. Assuming a cumulative dropout of 30% over this period and an additional 20% loss before completion of the first questionnaire, we expect an overall attrition rate of 50% by the fifth MTP. Consequently, a minimum of 320 participants must be enrolled to ensure that at least 160 of them provide valid data for matching at the fifth MTP. This would allow the primary hypothesis to be tested with high statistical power (0.95) in the overall study sample.

To examine the effectiveness of psychosomatic rehabilitation within relevant subgroups with a statistical power of 0.80, at least 80 matched participants with valid pre- and post-intervention data are required. Accounting for an expected matching loss of 20% and an overall attrition rate of 50% by the average post-intervention MTB increases the required subgroup size to 200 participants. Assuming that a relevant subgroup comprises at least one fifth of the total sample, a sample size of 1,000 participants (200 * 5) is required to ensure adequate statistical power for the subgroup analyses. Based on these calculations, the target sample size was set at 1,000 participants providing their informed consent.

### Recruitment

Recruitment was carried out by three DRV agencies: DRV Baden-Württemberg, DRV Mitteldeutschland (regional pension insurance agencies), and DRV Bund (the federal pension insurance agency), which participate in the project as collaborating partners. To ensure that participants met the inclusion criterion of completing at least one questionnaire before the start of their rehabilitation, recruitment had to take place shortly after rehabilitation approval. Therefore, it was planned that, at the end of each month, the participating DRV agencies would invite all eligible individuals whose psychosomatic rehabilitation was approved during the preceding two-week period to participate in the study. With regard to this period, a pilot assessment conducted prior to recruitment indicated that the participating DRV agencies approved psychosomatic rehabilitation for approximately 5,000 individuals. Thus, a recruitment period of up to six months, corresponding to up to six invitation waves, should be sufficient to achieve the target sample size of 1,000 participants.

The invitations to participate in the study include a cover letter, information about the study, an informed consent form, and a pre-addressed, postage-paid return envelope. After providing written informed consent, participants receive email invitations containing a link to the respective online questionnaire at six-week intervals. The first questionnaire is expected to take 10 to 15 min to complete, while the subsequent questionnaires are much shorter and should take half as long. As an incentive, participants who complete at least 9 of the 12 MTPs will receive €100. All participants who complete at least one questionnaire also enter a prize draw with the possibility of winning €1,000.

### Intervention

The intervention evaluated in this study is inpatient psychosomatic rehabilitation for individuals with mental or psychosomatic disorders funded by the DRV. This type of rehabilitation is provided by approximately 180 specialized psychosomatic rehabilitation clinics across Germany, with a total capacity of around 18,000 beds [34]. The standard duration is five weeks, which may be shortened or extended by a week in justified cases. Treatment is based on the biopsychosocial model of functioning and disability as reflected in the International Classification of Functioning, Disability and Health (ICF) [35, 36]. Accordingly, treatment addresses not only the underlying disorder but also its impact on social and occupational participation, taking into account the environmental and personal factors that influence functioning and disability [36].

The rehabilitation goals, which are explained to the patients at the beginning of the rehabilitation, are to reduce symptoms, restore functions, and/or improve capacities that are essential for social and occupational participation [35, 37]. Achieving these goals requires a multimodal treatment approach involving close collaboration among different professional groups, including physicians, therapists, and social workers [38].

Psychosomatic rehabilitation funded by the DRV is subject to a comprehensive quality assurance procedure. As part of this, the Rehabilitation Therapy Standards (RTSs) specify mandatory evidence-based treatment modules and their minimum scope of implementation (e.g., minimum treatment duration and minimum proportion of patients receiving a given treatment module). For psychosomatic rehabilitation, these modules include, among others, individual and group psychotherapy, patient education, exercise therapy, relaxation techniques, creative ergo- and art therapy, and services supporting social and occupational reintegration [39]. The RTSs further specify which interventions can be implemented within each module. For example, the relaxation module includes interventions such as biofeedback, autogenic training, progressive muscle relaxation, and mindfulness-based exercises. It is important to note that, with regard to the core psychotherapy modules, the current standards do not prescribe a specific therapeutic orientation. Consequently, psychosomatic rehabilitation clinics may employ different approaches, including psychodynamic and cognitive-behavioral therapy. These approaches were explicitly listed in the previous version of the RTSs, which remain applicable until the end of 2026 [40]. Thus, although specific treatment plans and interventions may differ between psychosomatic rehabilitation clinics, the RTSs provide a consistent, binding framework for the implementation of inpatient psychosomatic rehabilitation funded by the DRV.

**Fig. 1:**
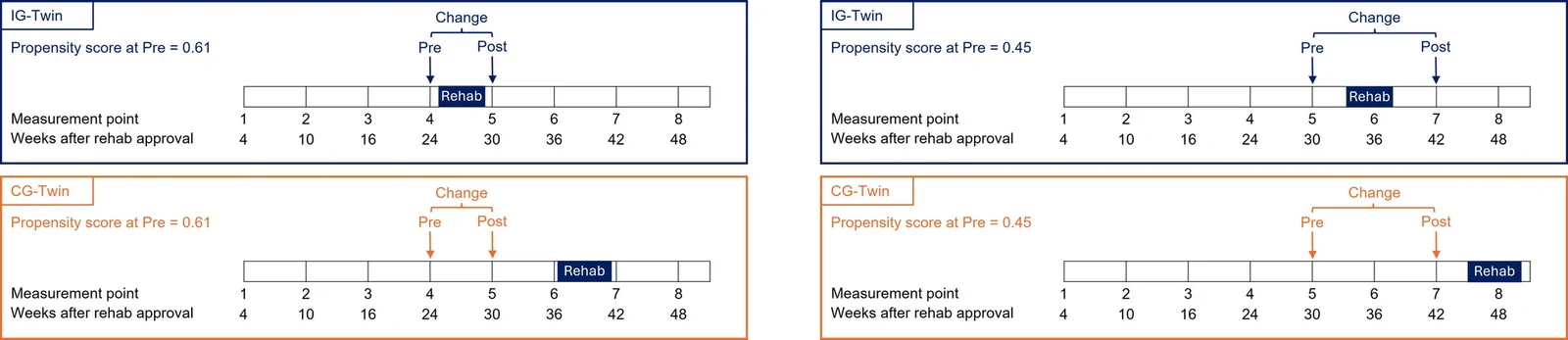
Examples of matched pairs to form the intervention group (IG) and the waitlist control group (CG)

The intervention evaluated in the present study is inpatient psychosomatic rehabilitation as implemented in routine care within the framework described above. The assignment to the IG or CG in the present study depends on the timing of the rehabilitation and the result of the matching procedure. The IG will comprise participants who undergo psychosomatic rehabilitation between the pre- and post-measurement point, whereas the CG will consist of matched participants whose rehabilitation has not yet begun by the post-measurement point (Fig. 1).

### Measures

Table 1 provides an overview of the outcomes and covariates included in this study. For each construct, it indicates the MTPs at which it is assessed, how it is assessed, and whether it is assessed via an online questionnaire or obtained from the DRV routine data.

**Table 1:** Measurement of outcomes and covariatesNext to KOMO in the line "Comorbidities" is where citation 54 is listed. This, therefore, addresses comment Q8: „The citation 54 seems to be missing in the manuscript“. Although citation 54 is not listed in the body of the text, it is included in this table.The same applies to citations 55 and 56, which are the subject of queries Q9 and Q10. The corresponding citations could be found in the row Psychotherapy motivation next to the FPTM-23 Initiative Scale and one row below next to MacArthur Scale.

Construct	Operationalization	Online questionnaire		Routine data from DRV
		T1	T2–T12	
**Primary outcome**				
Mental well-being	HEALTH-49, Module A [31]	x	x	
**Secondary outcomes**				
Work Ability	Work Ability Score [43]	x	x	
	Sick leave days during the last 6 weeks [42]	x	x	
Return to work	Employment situation	x	x	
	Continuous employment without being unable to work^1^			x
**Covariates**				
Disorder-related characteristics	Mental diagnosis^2^, year of first mental diagnosis^3^, use of disorder-related care services other than the rehabilitation^4,5,6^	x	x	x
Comorbidities	KOMO^7^ [54]	x		
Information about rehabilitation	Choice of clinic in application for rehabilitation^8^, changes in choice of clinic^8^, waiting time^9^	x	x	x
Psychotherapy motivation	FPTM-23 Initiative Scale [55]	x		
Sociodemographic attributes	Age^3^, gender^3^, MacArthur Scale [56]^3^, ethnicity^10^, education^10^	x		x

Notes: ^1^ Measured monthly, ^2^ primary diagnoses for the rehabilitation application coded with ICD-10 codes in the routine data ^3^ measured only with questionnaire at T1, ^4^ measured with questionnaires (T1–T12), ^5^ checklist of eight services such as outpatient psychotherapy, online services, self-help groups or stay in psychiatric acute clinic, ^6^ after rehabilitation has been completed, the checklist comprises two common post-rehabilitation follow-up care services. ^7^ Items on depressiveness/anxiety and dementia were omitted, as the former are our primary conditions (and thus not comorbidities) and the latter are extremely rare within our target group. ^8^ Measured with questionnaires (T1–T12) until the rehabilitation has begun, ^9^ calculated by date of approval and start date of rehabilitation, ^10^ only from routine data

#### Primary outcome

The primary outcome, mental well-being, is operationalized by the overall score of Module A of the HEALTH-49 questionnaire [31]. This module comprises 18 items that assess the mental well-being during the preceding two weeks across the three dimensions of somatoform complaints (7 items), depressiveness (6 items), and phobic anxiety (5 items). The three corresponding subscales have demonstrated good internal consistency (Cronbach’s α ≥ 0.82) [31, 41]. The convergent validity was supported by a high correlation between the overall score of Module A and the Global Severity Index of a short version of the Symptom Checklist-90-Revised (SCL-14; *r* = 0.89), and by high correlations (*r* > 0.80) of the three subscales with their corresponding SCL-14 subscales [22, 31]. In addition, both the overall score of Module A and its three subscales have demonstrated good sensitivity to change in studies with patients undergoing inpatient psychosomatic rehabilitation [16, 31, 33].

#### Secondary outcomes

Return to work after rehabilitation is defined as continuous employment subject to social insurance contributions without sickness absence for at least four consecutive weeks. This outcome will be operationalized in two ways. The first operationalization combines routine data provided by the DRV on monthly employment status with questionnaire data on sickness absence during the preceding six weeks. Although this operationalization incorporates objective employment data, the different reference periods of the two data sources may prevent an unambiguous determination of return to work for some observations, resulting in missing values. Therefore, we employ a second operationalization that is expected to have fewer missing values because it relies entirely on questionnaire data. This operationalization combines the current employment status with sickness absence during the preceding six weeks. As this operationalization uses a six-week rather than a one-month reference period, it is a more conservative measure of return to work.

Work ability is measured using two indicators of the Work Ability Index (WAI; [42]): the number of sick leave days during the preceding six weeks (i.e., since the previous MTP) and the Work Ability Score (WAS [43]). Both indicators are measured at each MTP. The WAS consists of a single item in which participants are asked to compare their current with their best ever work ability. Responses are recorded on an 11-point scale ranging from 0 (completely unable to work) to 10 (currently the best work ability). Previous research has shown that the WAS is a valid proxy for the full WAI [43, 44], making it a brief and efficient measure of work ability.

### Statistical analyses

We will examine our hypotheses using two complementary analytical approaches: a waitlist control group comparison (WCGC) with propensity score matching and a discontinuous growth model (DGM). The use of two analytical approaches is due to specific circumstances of the intervention under investigation and the resulting available data. For example, the preferred WCGC does not permit an evaluation of the medium-term effects of rehabilitation because waiting times until the start of rehabilitation are limited.

The WCGC is designed to estimate the causal effect of rehabilitation. With the use of propensity score matching, the aim of this approach is to approximate the design of a randomized controlled trial (RCT; [45]), thereby strengthening the internal validity of the findings. However, excluding participants during the matching process may reduce the external validity. In addition, this approach includes only two of the up to 12 measurement points of each participant, and—as already mentioned—only allows the examination of short-term effects of rehabilitation. Complementarily, the DGM allows the examination of short- and medium-term effects. By including all measurement points of each participant, this model-based approach uses the available data efficiently and allows rehabilitation effects to be estimated at predefined follow-up time points, despite substantial variation among participants in the timing of their follow-up MTPs (which is expected due to their individual rehabilitation dates). Because the DGM is employed on the overall sample (i.e., all participants are included), it will probably yield a higher external validity than the WCGC [46]. With respect to the short-term effect of rehabilitation, which can be examined with both approaches, the WCGC will serve as the main analysis and the DGM as the sensitivity analysis (Table 2). The medium-term effects can only be examined with the DGM. This approach allows a decomposition of the overall effect into the immediate impact at the completion of rehabilitation and the subsequent change during the post-rehabilitation phase up to the respective follow-up time point. This decomposition provides a differentiated view on the medium-term effectiveness of psychosomatic rehabilitation. It also allows the additional moderator analyses to examine moderator effects separately for the immediate rehabilitation effect and for the changes during the post-rehabilitation phase within the same model. In the following, we describe the procedures and statistical models of both approaches (WCGC and DGM).

#### Waitlist control group comparison with propensity score matching

This analytical approach consists of two steps: first, a matching procedure, and second, the analysis performed on the resulting matched sample.

##### Propensity score matching

Due to the longitudinal study design, participants can contribute observations to both the IG and the CG. Inclusion in the IG requires two MTPs between which the rehabilitation had taken place, whereas inclusion in the CG requires two MTPs during which the rehabilitation had not yet taken place. To ensure that each participant contributes to the analysis only once, a 1:1 propensity score matching will be applied. To identify eligible pairs for the matching, only observations with comparable temporal distances between rehabilitation approval and the corresponding pre- and post-MTP will be considered (Fig. 1). Accordingly, matched pairs will be restricted to observations with a similar timing of measurements relative to rehabilitation approval. Because of this temporal restriction, only a limited number of potential matches will be available for each participant.

Within this set of eligible matches, propensity scores will be estimated based on all measured variables known, or suspected, to be associated with the primary outcome (e.g., age, sex, diagnosis, duration of illness, and sick leave duration prior to rehabilitation). For each eligible pre-MTP, a propensity score will be estimated. Subsequently, propensity score differences will be calculated for all eligible matches. Matching will then be performed using nearest-neighbor matching (closest distance) within a caliper without replacement [47]. The caliper width will be set at 0.5 SD of the logit of the propensity score [48]. Among all eligible matches within this caliper width, matched pairs with the smallest propensity score distance will be iteratively selected. As a consequence, the assignment of participants to the IG or CG will be determined by the matching procedure and the resulting propensity score distances.

##### Waitlist control group comparison

In the subsequent analysis, the outcome at the post-rehabilitation MTP will be compared between the IG and the CG using an ANCOVA, with pre-rehabilitation values of the outcome included as a covariate (Table 2) [49]. By adjusting for pre-rehabilitation outcome values, this model tests whether the change from the pre- to the post-MTP differs between the two groups. As this model is applied to the matched sample consisting of matched pairs, pair membership will be included in the model as a fixed effect.

**Table 2:** Analytical approaches to examine the effects of psychosomatic rehabilitation

Analytical approach	Procedure and statistical model	Test	Effects of psychosomatic rehabilitation after		
			0 Month(Pre*→*Post)	3 Months (Pre*→*Follow-Up)	6 Months (Pre *→*Follow-Up)
Waitlist control group comparison with propensity score matching	1. Matching ─ 1:1 matching on patients’ propensity scores ─ Similar timing of measurement points relative to rehabilitation approval ─ Nearest-neighbor matching within a caliper without replacement 2. Paired analyses with matched sample (2 measurement points) $\:{Y}_{i}^{Post}={\beta\:}_{0}+\:{\beta\:}_{1}{GROUP}_{i}+{\beta\:}_{2}{Y}_{i}^{Pre}+{PAIR}_{i}\:+\:{\epsilon\:}_{i}$	Parameter	$\:{\beta\:}_{1}$	-	-
		Priority	Main analysis	-	-
Discontinuous growth model	1. Analyses with overall sample (all measurement points) Level 1: *Y*_*ti*_ *= π*_*0i*_ *+ π*_*1i*_*TIME*_*ti*_ *+ π*_*2i*_*REHA*_*ti*_ *+ π*_*3i*_*TIME.POST*_*ti*_ *+ π*_*4i*_*TVC*_*ti*_*+*$$\:{\epsilon\:}_{ti}$$ Level 2:*π*_0*i =*_$$\:\beta\:$$_00_*+ r*_0*i*_ *π*_1*i =*_$$\:\beta\:$$_10_*+ r*_1*i*_ *π*_2*i =*_$$\:\beta\:$$_20_*+ r*_2*i*_ *π*_3*i =*_ $$\:\beta\:$$_30_ *+ r*_3*i*_ *π*_4*i =*_ $$\:\beta\:$$_40_ *+ r*_4*i*_	Parameter	$$\:\beta\:$$_20_	$$\:{\beta\:}_{20}+\:{3\beta\:}_{30}\:$$	$$\:{\beta\:}_{20}+{6\beta\:}_{30}$$$$\:{\beta\:}_{20}+\:{6\beta\:}_{30}$$
		Priority	Sensitivity analysis	Secondary analysis	Secondary analysis

Notes. *Y* = outcome, outcomes of the waitlist control group comparison: mental well-being (primary outcome), work ability (secondary outcome). Outcomes of the discontinuous growth model: mental well-being, return to work (only for pre*→*follow-up effects), work ability.$\:{Y}^{Post}$= outcome at first measurement point after rehabilitation,$\:{Y}^{Pre}$= outcome at last measurement point before rehabilitation. *GROUP* = intervention group (rehabilitation between pre- and post-measurement point) vs. control group (no rehabilitation until post-measurement point). *PAIR* = dummy variables for matched pairs,$\:\beta\:$= fixed effects,$\:\epsilon\:$= Level-1 residuum, *r* = random effects. *TIME* = month since approval of rehabilitation (0 = approval of rehabilitation). Months were recorded to the exact day, with 1 month considered to be 30 days. *REHA* = rehabilitation completed, *TIME.POST* = month after end of rehabilitation (0 = until the end of the rehabilitation). *TVC* = time-varying covariates (person mean centered). Measurement points during rehabilitation are not included in the analyses, - examination not possible

#### Discontinuous growth model

This analytical approach will use a multilevel analysis (i.e., a linear mixed-effects model) comprising two levels (MTPs nested in participants). As shown in Table 2, the model specifies a linear change in the outcome over time (TIME). In addition, it estimates a shift in the outcome level in association with rehabilitation (REHA) and allows the post-rehabilitation trajectory to differ from the pre-rehabilitation trajectory (TIME.POST). Because measurements are taken every six weeks, patients could contribute one observation during rehabilitation at most, as inpatient rehabilitation usually takes five weeks (+/- 1 week). As a linear slope cannot be estimated from a single observation, the time during which patients undergo rehabilitation and, if present, the one possible measurement of the outcome during this time will be excluded from the analysis (but will be reported descriptively). Accordingly, the rehabilitation period is modeled as a discontinuity in the longitudinal course, represented by an immediate level shift in the outcome. The model further includes time-varying covariates (TVCs, person mean centered). In line with recommendations regarding the maximal random-effects structure for linear mixed-effects models, the model includes a random intercept (r_0*i*_) as well as random slopes (r_1*i*_ to r_4*i*_) for all Level-1 predictors [50, 51].

In this model, the short-term (immediate) effect of psychosomatic rehabilitation is captured by the fixed effect $\:\beta\:$_20_. The medium-term effects at three and six months after the completion of rehabilitation will be examined with linear contrasts of the fixed effects $\:{\beta\:}_{20}+\:{3\beta\:}_{30}\:$and $\:{\beta\:}_{20}+\:{6\beta\:}_{30}$, respectively. These contrasts represent the combined effect of the immediate change associated with rehabilitation and the subsequent change during the post-rehabilitation phase up to the respective follow-up time point.

Because this model focuses on within-person change over time, time-invariant covariates (TICs), such as sex, are not included in the primary analysis, as they do not explain within-person variation in the outcomes. To examine whether changes in the outcomes differ according to TICs, the latter will be incorporated (grand mean centered) into the Level-2 equations for *π*_0*i*_ to *π*_3*i*_. This specification allows moderator effects to be examined separately for the pre-rehabilitation, immediate rehabilitation, and post-rehabilitation phases.

#### Exploratory analyses

Exploratory analyses of the effectiveness of psychosomatic rehabilitation within relevant subgroups will be conducted by applying the analytical approaches separately within each subgroup, given that subgroup sample sizes are sufficiently large. To evaluate the effectiveness of psychosomatic rehabilitation across outcome dimensions, the analyses will additionally be performed for somatoform complaints, depressiveness, and phobic anxiety instead of overall mental well-being.

Moderator effects will be examined separately for each potential moderator by extending the respective analysis models accordingly. For example, to examine the moderating role of age, the WCGC will be extended to include age and its interaction with rehabilitation status (AGE x GROUP). In the DGM, moderation will be examined by extending the Level-2 equations for *π*_0*i*_ to *π*_3*i*_ to include the respective moderator, using age as an example. This enables the estimation of the moderator effects of age on both the pre-post rehabilitation effect (AGE x REHA) and the post-rehabilitation trajectory (AGE x TIME.POST).

Whether rehabilitation-related factors and patient-related factors affect the waiting time until rehabilitation will be examined for all prespecified predictors simultaneously using parametric time-to-event analyses (accelerated failure time models). In addition to testing the significance of the predictors, these models allow an estimation of the average difference in waiting time associated with specific predictors (e.g., making use of the right to choose a rehabilitation clinic vs. not making use of this right), expressed as the average number of days (longer or shorter) until rehabilitation initiation.

## Discussion

Inpatient psychosomatic rehabilitation is a central component of routine care for individuals with chronic mental and psychosomatic disorders in Germany aimed at improving functioning and promoting social and occupational participation. By including a waitlist control group and combining two complementary analytical approaches (WCGC and DGM), the present study will provide comparatively robust evidence on the effectiveness of psychosomatic rehabilitation in routine care with respect to mental well-being, work ability, and return to work.

In addition, the subgroup and moderator analyses will provide a more differentiated understanding of whether—and if so, the extent to which—the effectiveness of psychosomatic rehabilitation varies across patient-related characteristics and contextual factors, both with respect to the immediate rehabilitation effect and during the subsequent post-rehabilitation phase. A better understanding of which patients benefit more or less during the rehabilitation and post-rehabilitation phases may provide a basis for evaluating and optimizing rehabilitation interventions and follow-up care—for example, with the aim of promoting more equitable rehabilitation outcomes regarding sex and educational level.

Furthermore, by modeling changes in mental well-being during the waiting period and examining determinants of waiting time until rehabilitation, the present study sheds light on clinically pertinent and health service–relevant aspects of psychosomatic rehabilitation that have received comparatively little empirical attention despite their recognized importance [29]. The identification of factors associated with waiting time may provide a basis for optimizing referral and allocation processes and, ultimately, reducing waiting times.

### Strengths and limitations

One major strength of the present study is the inclusion of a waitlist control group. The corresponding waitlist control group comparison with propensity score matching provides higher internal validity and allows for stronger causal inferences regarding the effectiveness of psychosomatic rehabilitation than previous studies, which have predominantly relied on uncontrolled pre-post designs. Another strength is the combination of patient-reported questionnaire data with DRV routine data. While the questionnaires capture outcomes that are best assessed from the patient’s perspective, such as mental well-being and perceived work ability, the routine data provide objective information on the return-to-work outcome, as well as important background information, such as sick leave during the year prior to rehabilitation and the primary diagnosis underlying rehabilitation approval. Combining both data sources enables a more comprehensive evaluation of rehabilitation effectiveness than would be possible using either source alone. Finally, the recruitment strategy minimizes selection bias at the invitation stage, as all individuals whose application for psychosomatic rehabilitation was approved by one of the participating DRV agencies were invited to participate. As self-selection bias due to voluntary participation cannot be completely excluded, the representativeness of the study sample will be evaluated by comparing participants, based on DRV routine data, with the entire population of individuals approved for psychosomatic rehabilitation by the participating DRV agencies in 2026. These agencies cover more than half of all individuals insured by the German statutory pension insurance system before retirement [52], thereby providing a broad basis for recruitment. Nevertheless, because the study is limited to beneficiaries of these three agencies, the findings may not be fully generalizable to all rehabilitation recipients insured by the German statutory pension insurance system.

One major limitation of the present study is that, due to the chosen study design, only the short- and medium-term effects of psychosomatic rehabilitation can be examined, while long-term effects beyond six months after rehabilitation cannot be evaluated. Furthermore, although propensity score matching can reduce bias due to unmeasured confounding when unmeasured confounders are associated with the observed covariates [53], residual confounding cannot be completely excluded due to the observational nature of the study. Finally, as MTPs are anchored to study enrollment rather than to rehabilitation, the pre- and post-measurements do not necessarily coincide with the start and completion of rehabilitation. However, these differences are expected to be small (3 weeks on average) and are addressed through both the matching procedure and statistical adjustment.

## Data Availability

No datasets were generated or analysed during the current study.
